# Supplementary material for: The relationship between chronotype and intelligence: the importance of work timing
Source: Sci Rep. 2020 Apr 28;10:7105. doi: 10.1038/s41598-020-62917-9 (PMC7188801; doi:10.1038/s41598-020-62917-9)
Supplement: Supplementary file 1 — Supplementary information. [file 41598_2020_62917_MOESM1_ESM.pdf]

The relationship between chronotype and intelligence: the importance of work timing

Péter P. Ujma[1,2], Tanja G. Baudson[3, 4], Róbert Bódizs[1,2], Martin Dresler[5]

1 Institute of Behavioural Sciences, Semmelweis University, Budapest, Hungary

2 National Institute of Clinical Neuroscience, Budapest, Hungary

3 Mensa in Germany, Cham, Germany

4 Institute for Globally Distributed Open Research and Education (IGDORE)

5 Donders Institute for Brain, Cognition and Behaviour, Radboud University Medical Centre, Nijmegen, Netherlands

Correspondence:

Róbert Bódizs, bodizs.robert@med.semmelweis-univ.hu

Supplementary table S1. Descriptive statistics for covariates.

		N	Minimum	Maximum	Mean	SD
Mensa members	Work start time	481	04:00:00	15:00:00	08:35:23	01:18:15
	Commute duration	483	0.0	120.0	22.8	19.6
	Cigarettes/day	515	0.0	40.0	1.2	4.6
	Beer/day	515	0.0	12.6	0.3	0.8
	Glass wine/day	515	0.0	4.3	0.2	0.5
	Liquor/day	515	0.0	2.0	0.1	0.2
	Coffee/day	515	0.0	13.6	1.5	1.8
	Caffeine drink/day	515	0.0	14.3	0.4	1.3
	Tea/day	515	0.0	14.3	0.6	1.6
	Hypnotic/day	515	0.0	1.0	0.0	0.2
Controls	Work start time	272	5:30:00	16:00:00	8:21:23	1:21:06
	Commute duration	275	0.0	100.0	24.2	18.8
	Cigarettes/day	293	0.0	28.6	2.5	5.8
	Beer/day	294	0.0	14.3	0.4	1.1
	Glass wine/day	294	0.0	2.9	0.3	0.4
	Liquor/day	294	0.0	1.4	0.1	0.2
	Coffee/day	294	0.0	10.0	1.9	1.7
	Caffeine drink/day	294	0.0	7.1	0.3	0.8
	Tea/day	294	0.0	7.1	0.5	1.0
	Hypnotic/day	294	0.0	1.0	0.0	0.1

Supplementary table S2. Pearson's correlation coefficients between covariates.

		Work start time	Commute duration	Cigarettes/day	Beer/day	Glass wine/day	Liquor/day	Coffee/day	Caffeine drink/day	Tea/day	Hypnotic/day
Work start time	r	1	-.051	-.063	-.007	.021	-.010	-0.105	.046	.065	.069
	p		.162	.087	.854	.560	.793	.004	.209	.077	.060
	N	753	753	746	747	747	747	747	747	747	747
Commute duration	r	-.051	1	-.006	-.023	-.035	-.041	-.002	-0.072	-.010	-.005
	p	.162		.863	.528	.338	.262	.953	.048	.779	.892
	N	753	758	751	752	752	752	752	752	752	752
Cigarettes/day	r	-.063	-.006	1	0.331	.062	.010	0.234	0.136	-.065	-.011
	p	.087	.863		.000	.079	.768	.000	.000	.066	.754
	N	746	751	808	808	808	808	808	808	808	808
Beer/day	r	-.007	-.023	0.331	1	0.079	0.174	0.224	-.039	-.021	.019
	p	.854	.528	.000		.025	.000	.000	.262	.559	.587
	N	747	752	808	809	809	809	809	809	809	809
Glass wine/day	r	.021	-.035	.062	.079*	1	.062	0.186	-0.104	0.091	.039
	p	.560	.338	.079	.025		.079	.000	.003	.010	.270
	N	747	752	808	809	809	809	809	809	809	809
Liquor/day	r	-.010	-.041	.010	0.174	.062	1	.019	-.033	-.024	-.005
	p	.793	.262	.768	.000	.079		.592	.354	.500	.894
	N	747	752	808	809	809	809	809	809	809	809
Coffee/day	r	-0.105	-.002	0.234	0.224	0.186	.019	1	-0.117	-.055	-0.082
	p	.004	.953	.000	.000	.000	.592		.001	.117	.020
	N	747	752	808	809	809	809	809	809	809	809
Caffeine drink/day	r	.046	-0.072	.136	-.039	-0.104	-.033	-0.117	1	-.044	.043
	p	.209	.048	.000	.262	.003	.354	.001		.215	.225
	N	747	752	808	809	809	809	809	809	809	809
Tea/day	r	.065	-.010	-.065	-.021	.091**	-.024	-.055	-.044	1	-.012
	p	.077	.779	.066	.559	.010	.500	.117	.215		.729
	N	747	752	808	809	809	809	809	809	809	809
Hypnotic/day	r	.069	-.005	-.011	.019	.039	-.005	-0.082	.043	-.012	1
	p	.060	.892	.754	.587	.270	.894	.020	.225	.729	
	N	747	752	808	809	809	809	809	809	809	809
